# Adaptive Super-Twisting Observer for Estimation of Random Road Excitation Profile in Automotive Suspension Systems

**DOI:** 10.1155/2014/203416

**Published:** 2014-02-09

**Authors:** J. J. Rath, K. C. Veluvolu, M. Defoort

**Affiliations:** ^1^School of Electronics Engineering, Kyungpook National University, Daegu 702-701, Republic of Korea; ^2^LAMIH, CNRS UMR 8201, University Lille Nord de France, UVHC, 59313 Valenciennes, France

## Abstract

The estimation of road excitation profile is important for evaluation of vehicle stability and vehicle suspension performance for autonomous vehicle control systems. In this work, the nonlinear dynamics of the active automotive system that is excited by the unknown road excitation profile are considered for modeling. To address the issue of estimation of road profile, we develop an adaptive supertwisting observer for state and unknown road profile estimation. Under Lipschitz conditions for the nonlinear functions, the convergence of the estimation error is proven. Simulation results with Ford Fiesta MK2 demonstrate the effectiveness of the proposed observer for state and unknown input estimation for nonlinear active suspension system.

## 1. Introduction

The automotive vehicle suspension dynamics contribute significantly in evaluating the effective performance with regard to passenger comfort, road handling, and stability of the vehicle [1]. The design of suspension systems evolved from passive suspensions to the active suspension system [2] adding more control capabilities. Suspension dynamics predominantly provide information regarding vertical stability [2, 3] of the vehicle. Effective analysis of the suspension performance provides information regarding the vertical load acting on the vehicle, a critical component in determining the effective tractive force [3]. In such scenarios, the suspension system dynamics that replicate the behavior of mass spring damper system [3] are complex to analyze when nonlinear behavior of the spring and the damper systems are considered. Road profile that replicates the randomness of road surface in form of cleats or troughs affects the suspension system performance [4]. The different levels of road excitation necessitate the continuous regulation of damping force generated by the suspension for maintaining the stability of the vehicle. As the vehicle operating range varies, analysis of the nonlinear dynamics of the suspension system excited by the road profile is one of the major domains of research for suspension systems.

Road profile is a critical parameter that results in undesirable vertical vibrations for the vehicle if it is not compensated by an adequate control effort in the suspension. These unfavorable vibrations result in dynamic variations in the vertical load of the vehicle affecting its stability. The design of suitable controllers to compensate for these variations is dependent on the effective measurement of the road profile. For experimental purposes, expensive instruments called profilographs are used for measuring the road profile [4, 5]. In [6], a real time conditioning algorithm was designed to measure the road profile based on measurement of the vertical acceleration. As an alternative to these expensive instrumentation and sensor technology that are affected by noise, estimation of the road profile by use of observers has been an important issue.

In [7], a Kalman filter-based estimation of the road profile measured using a road profilometer was performed. The designed estimation worked on the vertical dynamics of an active suspension system and was experimentally validated. In [8], a neural network-based approach was adopted to estimate the random road profile. This work employed accelerometers to estimate the road profile modeled as a function of road roughness coefficient. In [9], a minimum order observer was designed for a linearized model of passive suspension dynamics to estimate the road profile.

Sliding mode theory [10, 11] has evolved over time as an effective tool for estimation of unknown inputs in control domain. In [12], a first order sliding mode observer was designed to estimate the states of the suspension system under the influence of different road profiles. To remove the chattering effect that is inherent in first order sliding mode [13–17], higher order sliding mode theory was developed. Recently, HOSM observers have been popular for state and unknown input estimation in uncertain nonlinear systems [18–20]. For systems with relative degree one, the super-twisting algorithm (STA) served as an ideal tool for the estimation of unknown inputs [21]. In [22–24], the STA based observer was used to estimate the road profile acting as unknown input to the system. By measurement of the vertical velocities, the road profile and the tire forces were estimated. All these works [7–9] did not consider the nonlinear dynamics of the suspension system. Other works [22–24] did not consider the randomness of the road profile.

To address these issues, we consider the nonlinear dynamics of the active suspension system for a quarter vehicle excited by a random road profile. The suspension dynamics considered in this paper effectively replicate the nonlinear behavior of the spring and damper of the suspension. The road excitation profile is considered as an unknown input in this work and is estimated with an adaptive STA observer [25]. For analysis, the road roughness values are based on power spectral density (PSD) values as proposed by International Organization for Standardization (ISO) [4, 5]. For the design of the observer, under the rank conditions for the output matrix, the system is then partitioned into two subsystems where the unknown input appears in one subsystem. For the subsystem affected by the unknown input, an adaptive STA based observer is then designed to ensure the stability of the error dynamics of the subsystem in finite time. For the subsystem without unknown inputs, a nonlinear observer is designed under Lipschitz conditions to ensure the stability of the system in sliding mode. The application of the proposed method to the modelled vehicle dynamics is validated through simulations.

Throughout this paper, *λ*_max⁡_(**A**) denotes the maximum eigenvalue of matrix **A**, ||**A**|| denotes the 2-norm $\sqrt{\lambda_{\max}(A^{T}A)}$ of a matrix **A**, and *σ*_min⁡_(**A**) represents the minimum singular value of matrix **A**. For any vector *z* = [*z*_1_,…, *z*_*q*_]^*T*^ ∈ *R* ^*q*^ and any scalar *α* ∈ *R*, we denote

(1) $$\begin{array}{c} \mathrm{sign}\left(z\right)={\left[\mathrm{sign}\left(z_{1}\right),\ldots ,\mathrm{sign}\left(z_{q}\right)\right]}^{T},\\ {\left|z\right|}^{\alpha }=\mathrm{diag}\left({\left|z_{1}\right|}^{\alpha },\ldots ,{\left|z_{q}\right|}^{\alpha }\right),\\ {\left\lceil z\right\rfloor }^{\alpha }={\left|z\right|}^{\alpha }\mathrm{sign}\left(z\right). \end{array}$$

## 2. Modeling Active Suspension Dynamics

**Figure 1:**
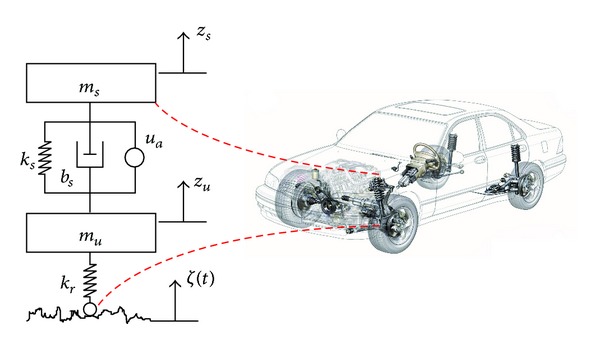
Active suspension system for a quarter wheel.

The active suspension system in vehicles incorporates an active controlled force actuator instead of the shock absorber generally found in a passive suspension system. The nonlinear dynamics that govern the active suspension system are given as [26]

(2) $$\begin{array}{c} m_{s}{\ddot{z}}_{s}+f_{k}+f_{b}=u, \end{array}$$

(3) $$\begin{array}{c} m_{u}{\ddot{z}}_{u}-f_{k}-f_{b}+k_{r}\left(z_{u}-\zeta \left(t\right)\right)=-u, \end{array}$$

where *m*_*s*_ is the sprung mass or the vehicle mass, *m*_*u*_ is the unsprung mass or the wheel mass, *z*_*s*_ is the sprung mass displacement, *z*_*u*_ is the unsprung mass displacement, *u* is the controlled actuator force, *k*_*r*_ is the tire stiffness, and *ζ*(*t*) is the road excitation profile. The nonlinear damping force, *f*_*b*_, and spring force, *f*_*k*_, for the suspension dynamics can be described as [26]

(4) $$\begin{array}{c} f_{k}=k_{s}\left(z_{s}-z_{u}\right)+k_{s\text{nl}}{\left(z_{s}-z_{u}\right)}^{3}, \end{array}$$

(5) $$\begin{array}{c} f_{b}=b_{s}\left({\dot{z}}_{s}-{\dot{z}}_{u}\right)+b_{s\text{nl}}\sqrt{\left|{\dot{z}}_{s}-{\dot{z}}_{u}\right|}\mathrm{sign}\left({\dot{z}}_{s}-{\dot{z}}_{u}\right), \end{array}$$

where *k*_*s*_ is the linear spring stiffness constant, *b*_*s*_ is the linear damper constant, *k*_*s*nl_ is the nonlinear spring stiffness, and *b*_*s*nl_ is the nonlinear damping constant. The motion of the vehicle over a bump that restricts the wheel travel within a given range and prevents contact between the tyre and the vehicle body is effectively modeled by the nonlinear spring force, *f*_*k*_. Similarly for the damper, the damping force generated while the wheel traverses in vertical direction owing to road profile is a nonlinear effect. This nonlinear effect is well approximated by the nonlinear dynamics as depicted in (5). In this work, the damping force provided by the tyre which is very complex to model has been neglected. The active suspension dynamics for a quarter wheel vehicle model together with the modeling parameters are shown in Figure 1. The active suspension dynamics are affected by the road profile requiring control of the effective damping force needed to be provided by the actuator for good handling of the vehicle [1], ride performance, and road stability. Road profile is often modeled as a sinusoidal disturbance or a trapezoidal disturbance to identify with crests/trough or cleats that appear on practical roads. This type of modeling however does not represent the typical roughness profile of roads and the resulting effects it has on the suspension. The standards of road roughness according to ISO [4, 5] can be classified into different road classes as shown in Table 1.

The integrated dynamics (2)–(5) for the active suspension system can be represented in state space as

(6) x˙=Ax+BΨ(x,u,t)+Eζ(t),y=Cx.

With

(7) x=[x1x2x3x4]T=[z˙uz˙szszu]T,A=[−bsmubsmuksmu−ks+krmubsms−bsms−ksmsksms01001000],  B=[1mu−1ms00],E=[krmu000],

where

(8) Ψ(x,u,t)=−u+ksnl(x3−x4)3+bsnl|x2−x1|sign⁡(x2−x1).

In the modeled system dynamics, the active actuator control force *u* is the control input for the system. In the system dynamics (6), the displacement of the sprung mass, *x*_3_, and velocity of the unsprung mass, *x*_1_, are considered as outputs, and the output matrix can be defined by

(9) $$\begin{array}{c} C=\left[\begin{array}{cccc} 1 & 0 & 0 & 0\\ 0 & 0 & 1 & 0 \end{array}\right]. \end{array}$$

The unknown input for the system is the road profile denoted as *ζ*(*t*). The measurement of road profile is an extremely complex task that requires the use of complex measuring instruments such as profilographs [4, 5] that are expensive and impractical. Hence our focus is on the development of an approach to estimate the random road profile, *ζ*(*t*), for the active suspension systems.

## 3. Observer Design

In this section, we discuss the design of the observer for the active suspension system. A combination of nonlinear Lipschitz observer and adaptive super-twisting observer is employed. To facilitate the design of the observer, the following assumptions are required.

**Assumption 1**

All invariant zeros of the triple (*A*, *E*, *C*) must lie in the left half plane and rank (*CE*) = rank (*E*).

**Assumption 2**

The nonlinear functions in Ψ(*x*, *u*, *t*) satisfiy the Lipschitz conditions.

**Assumption 3**

The function *ζ*(*t*) and its first derivative are bounded.

**Assumption 4**

The control input is bounded and the system is assumed to be bounded input bounded state stable (BIBS).

For the dynamics defined in (6), it can be easily verified that the rank⁡(*CE*) = rank⁡(*E*) = 1. Further, the triple (*A*, *E*, *C*) does not contain any invariant zeros that lie on the right hand plane. The nonlinear function Ψ(*x*, *u*, *t*) in the system dynamics (6) can be divided into Ψ_1_(*x*, *u*, *t*) and Ψ_2_(*x*, *u*, *t*) to analyze the Lipschitz continuity as follows:

(10) Ψ(x,u,t)=Ψ1(x,u,t)+Ψ2(x,u,t),Ψ1(x,u,t)=ksnl(x3−x4)3,Ψ2(x,u,t)=bsnl|x2−x1|sign⁡(x2−x1).

With *x*_3_, that is, *z*_*s*_ and *x*_4_, that is, *z*_*u*_ representing the physical states of practically realizable automotive suspension system, the function Ψ_1_(*x*, *u*, *t*) can be determined to be locally Lipschitz with a Lipschitz constant obtained as *l*_Ψ_1__ = *k*_*s*nl_ | *z*_*s*_ − *z*_*u*_|^2^. Similarly for the nonlinear function Ψ_2_(*x*, *u*, *t*), with *x*_2_, that is, ${\dot{z}}_{s}$ and *x*_1_, that is, ${\dot{z}}_{u}$ bounded, local Lipschitz continuity can be established. The Lipschitz constant for Ψ_2_(*x*, *u*, *t*) is obtained as $l_{\Psi_{2}}=\left(b_{s\text{nl}}/2\right)(1/{\left|{\dot{z}}_{s}-{\dot{z}}_{u}\right|}^{1/2})$. For the active automotive suspension system modeled in (6), by use of the actuator control force, *u*_*a*_, it can be asserted that $({\dot{z}}_{s}-{\dot{z}}_{u})\neq 0$ such that the function Ψ(*x*, *u*, *t*) maintains its Lipschitz continuity with a Lipschitz constant, *l*_*β*_ = *l*_Ψ_1__ + *l*_Ψ_2__.

In the modeled system dynamics (6), the unknown input *ζ*(*t*) is the road profile that is considered as function of road roughness coefficient and other physical parameters relating to the road conditions which are bounded. It can be thus deduced from the dynamics of the road profile model that the road excitation profile and its derivative are both bounded. For the modeled active suspension dynamics (6), the control input to the system is the actuator force, *u*, that is bounded.

In order to design the combined observer, the original system dynamics will be divided into two subsystems, such that one of the subsystems will be free from unknown inputs. With the Assumptions 1–4 for the active suspension system, (6) being satisfied, we can directly partition system (6) into two subsystems *S*1 and *S*2 as follows:

(11) $$\begin{array}{c} S1:\{\begin{array}{cc} {\dot{x}}_{1}=A_{11}x_{1}+A_{12}z+B_{1}\Psi \left(x,u,t\right)+E_{1}\zeta \left(t\right), & \\ y_{1}=C_{1}x_{1}, & \end{array} \end{array}$$

(12) $$\begin{array}{c} S2:\{\begin{array}{cc} {\dot{x}}_{2}=A_{21}x_{1}+A_{22}z+B_{2}\Psi \left(x,u,t\right), & \\ y_{2}=C_{2}z, & \end{array} \end{array}$$

where

(13) x1=x1,  z=[x1x2x3]T,C1=[1],  C2=[010],A11=[−bsmu],  A12=[bsmuksmu−ks+krmu]T,A21=[bsms01],  A22=[−bsms−ksmsksms100000],B1=[1mu],  B2=[−1ms00],  E1=[krmu].

**Figure 2:**
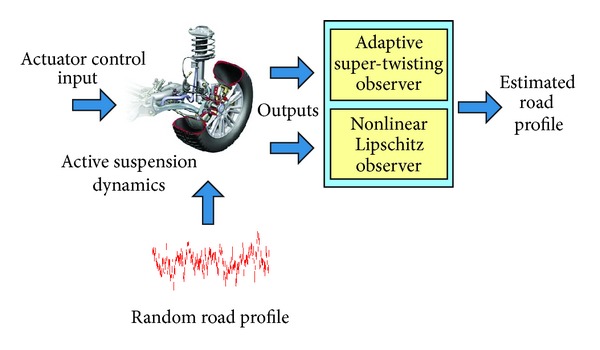
Overview of proposed observer.

With the system (6) partitioned as above, the objective is to design an adaptive STA based observer to estimate the states and unknown input for the *S*1-subsystem (11) and a nonlinear Lipschitz observer (NLO) to estimate the states for the *S*2-subsystem (12). The overview of the design is shown in Figure 2.

The estimation error can be defined as

(14) e=x^−x=[e1ez]T=[e1[e2e3e4]T]T,

where $\hat{x}$ is the observed state and *e*_1_ and *e*_*z*_ are the errors for the subsystems *S*1 and *S*2.

### 3.1. Adaptive Super-Twisting Observer Design for S1-Subsystem

For *S*1-subsystem (11) satisfying the above assumptions, the following observer based on the adaptive STA can be designed to estimate the states and the unknown input:

(15) $$\begin{array}{c} {\dot{\hat{x}}}_{1}=A_{11}{\hat{x}}_{1}+A_{12}\hat{z}+B_{1}\Psi \left(\hat{x},u,t\right)+E_{1}^{}\nu \left(t\right), \end{array}$$

where *ν*(*t*) is the robust sliding term based on the adaptive STA [25] and defined as

(16) $$\begin{array}{c} \nu \left(t\right)=-K_{1}{\left\lceil {\hat{x}}_{1}-x_{1}\right\rfloor }^{1/2}-K_{2}\int_{0}^{t}\mathrm{sign}\left({\hat{x}}_{1}-x_{1}\right). \end{array}$$

The adaptive gains *K*_1_, *K*_2_ in (16) are designed as

(17) K˙1={κ1sign⁡(|x^1−x1|−ϵ),if  K1>αm,κ2,if  K1≤αm,K2=κ3K1,

where *κ*_1_, *κ*_2_, *κ*_3_, and *ϵ* are positive constants. The parameter *α*_*m*_ is an arbitrary small positive constant.

To establish the convergence of the observer dynamics, the error dynamics (14) can be obtained as $e_{1}={\hat{x}}_{1}-x_{1}$, which serves as the sliding surface for the designed adaptive STA based observer. The objective of the designed observer is to ensure that the error converges to zero and to reconstruct the unknown road excitation profile, *ζ*(*t*), from the robust term (16).

**Theorem 5**

For system (11) satisfying the Assumptions 1–4, the observer system (15) with the robust term (16) will ensure that the error dynamics (*e*_1_) will converge to zero in finite time.

**Proof**

The error dynamics of the system (11) can be obtained from (11) and (15) as

(18) e˙1=A11e1+A12ez+B1Ψ(x^,u,t)−B1Ψ(x,u,t)  +ν(t)−E1ζ(t) =Λ(e1,ez,t)+ν(t),

where Λ(*e*_1_, *e*_*z*_, *t*) includes the perturbation terms. The matrices *A*_11_ and *A*_12_ are known, and hence they are bounded. As system satisfies Assumptions 2–4, the boundedness of the nonlinear function $\Psi (\hat{x},u,t)$ and the unknown input, *ζ*(*t*), can be easily established. For the obtained error dynamics (18), it can be proved that ${\dot{e}}_{1}$ is locally bounded by a constant, as *e*_1_ is twice differentiable on a compact set. This is not restrictive as the active suspension system dynamics are bounded at least locally. It will be shown later that the subsystem *S*2 is asymptotically stable. Further, as the system is free from unknown inputs, under the Assumptions 2–4, the subsystem *S*2 boundedness can be established. Based on the above arguments, the boundedness of the perturbation Λ(*e*_1_, *e*_*z*_, *t*) is obtained as

(19) $$\begin{array}{c} \dot{\Lambda }\left(e_{1},e_{z},t\right)\leq \rho , \end{array}$$

where *ρ* is a constant (not necessarily known). With the perturbation terms Λ(*e*_1_, *e*_*z*_, *t*), satisfying the condition (19) required for the adaptive STA (15), the convergence of the error dynamics (18) can now be proved with the following Lyapunov function:

(20) $$\begin{array}{c} V\left(e_{1}\right)=\Omega^{T}P\Omega +\frac{1}{2\tau_{1}}{\left(K_{1}-K_{1}^{*}\right)}^{2}+\frac{1}{2\tau_{2}}{\left(K_{2}-K_{2}^{*}\right)}^{2}, \end{array}$$

where $\Omega ={\left[\begin{array}{cc} {\left|e_{1}\right|}^{1/2}\mathrm{sign}(e_{1}) & e_{1} \end{array}\right]}^{T}$ and *τ*_1_, *τ*_2_, *K*_1_*, and *K*_2_* are positive constants and *P* is a positive definite matrix. Similar to the results in [25] with *K*_1_ and *K*_2_ satisfying (17), $\dot{V}(e_{1})$ can be shown to be a negative definite and the error converges to zero in finite time. The sliding surface is thus reached in finite time and maintained thereafter.

### 3.2. Nonlinear Lipschitz Observer for S2-Subsystem

For subsystem (12), a NLO is designed as follows to estimate the states of the system:

(21) $$\begin{array}{c} \dot{\hat{z}}=A_{21}x_{1}+A_{22}\hat{z}+B_{2}\Psi \left(\hat{x},u,t\right)+L\left(y_{2}-C_{2}z\right), \end{array}$$

where the feedback $L={\left[\begin{array}{ccc} l_{11} & l_{21} & l_{31} \end{array}\right]}^{T}$ is to be discussed in Theorem 6 later. The error dynamics (14) of the subsystem *S*2 can be obtained as

(22) [e˙2e˙3e˙4]=[−bsms−ksms−l11ksms1−l2100−l310]    +[−100](Ψ(x^,u,t)−Ψ(x,u,t)).

The following theorem establishes the stability of the *S*2-subsystem.

**Theorem 6**

For system (12) satisfying the Assumptions 1–4, the observer (21) ensures that the state estimation error (*e*_*z*_) is asymptotically stable provided that the gain L satisfies

(23) $$\begin{array}{c} Q\left(A_{22}-LC_{2}\right)+{\left(A_{22}-LC_{2}\right)}^{T}Q+l_{\beta }^{2}QQ+I<0, \end{array}$$

where *l*_*β*_ is the Lipschitz constant for Ψ(*x*, *u*, *t*) (Assumption 2) and Q is a positive definite matrix.

**Proof**

With the convergence of the subsystem *S*1 error (*e*_1_) to zero in the sliding mode, the error dynamics (14) can be written as

(24) $$\begin{array}{c} {\dot{e}}_{z}=\left(A_{22}-LC_{2}\right)+\left(\Psi \left(\hat{x},u,t\right)-\Psi \left(x,u,t\right)\right). \end{array}$$

With the system satisfying Assumption 2, the Lipschitz constant for Ψ(*x*, *u*, *t*) is evaluated as *l*_*β*_. With the choice of the Lyapunov function as *V*(*e*_*z*_) = *e*_*z*_^*T*^ *Qe*_*z*_ and differentiating with respect to time, one has

(25) V˙(ez)=ezT[(A22−LC2)TQ+Q(A22−LC2)]ez+2ezTQ(Ψ(x^,t)−Ψ(x,t)).

From the above results, its can be deduced that

(26) V˙(ez)≤ezT[(A22−LC2)TQ+Q(A22−LC2)]ez+2lβ||Qe||||ez||.

In the sliding mode as *e*_1_ = 0, we have

(27) $$\begin{array}{c} e={\left[\begin{array}{cc} 0 & e_{z} \end{array}\right]}^{T}={\left[\begin{array}{cc} 0 & {\left[\begin{array}{ccc} e_{2} & e_{3} & e_{4} \end{array}\right]}^{T} \end{array}\right]}^{T}. \end{array}$$

It can thus be written as follows:

(28) V˙(ez)≤ezT[(A22−LC2)TQ+Q(A22−LC2)]ez+2lβ||Qez||||ez||.

Further, one can obtain

(29) V˙(ez)≤ez((A22−LC2)TQ+Q(A22−LC2)+lβ2QQ+I)×ez,

where 2*l*_*β*_||*Qe*_*z*_||||*e*_*z*_|| ≤ (*l*_*β*_)^2^ *e*_*z*_ *Q* *Qe*_*z*_ + *e*_*z*_^*T*^ *e*_*z*_ is satisfied. If the design of the feedback gain, *L*, is such that (23) is satisfied, then $\dot{V}(e_{2})<0$. The error dynamics will thus be asymptotically stable.

**Remark 7**

Equation (23) can be written as an algebraic Riccati equation in the following form:

(30) $$\begin{array}{c} Q\left(A_{22}-LC_{2}\right)+{\left(A_{22}-LC_{2}\right)}^{T}Q+l_{\beta }^{2}QQ+I+\gamma I=0 \end{array}$$

for some *γ* > 0. The following condition [27, 28] ensures the asymptotic stability of the system (24):

(31) $$\begin{array}{c} \underset{w\in {\mathbb{R}}^{+}}{\min}\;\sigma_{\min}\left(A_{22}-LC_{2}-jwI\right)>l_{\beta }, \end{array}$$

where *σ*_min⁡_(·) represents the minimum singular value of a matrix. If the above condition (23) is satisfied and if there exists a stable (*A*_22_ − *LC*_2_) matrix, then there exists a symmetric positive definite (SPD) solution *Q* = *Q* ^*T*^ for the Riccati equation (30).

### 3.3. Estimation of Unknown Input: Road Excitation Profile

In the sliding mode, with the error *e*_1_ converging to zero in finite time $\left(e_{1}={\dot{e}}_{1}=0\right)$, the equivalent control [29] can be obtained from (18) as follows:

(32) $$\begin{array}{c} v_{\text{eq}}=-\Lambda \left(e_{1},e_{z},t\right)+E_{1}\zeta \left(t\right). \end{array}$$

As *e*_1_ → 0 in finite time, and the nonlinearities satisfy Lipschitz assumptions, we have from (18) the following:

(33) ||Λ(e1,ez,t)||≤(||A12−LC2||+lβ)||e(t)||⟶0(t⟶∞).

The unknown road excitation profile when *t* → *∞* can be thus obtained as

(34) $$\begin{array}{c} \hat{\zeta }\left(t\right)=E_{1}^{-1}K_{2}\int_{0}^{t}\mathrm{sign}\left(e_{1}\right)dt. \end{array}$$

**Remark 8**

The design of the adaptive STA observer and the NLO considered in this work can be easily extended for estimation of multiple unknown inputs. The general class of nonlinear systems that is similar to (6) is represented by

(35) x˙=Ax+BΨ(x,u,t)+Ef(t),y=Cx,

where *x* ∈ *M* ⊂ ℝ^*n*^, *A* ∈ ℝ^*n*×*n*^, *C* ∈ ℝ^*p*×*n*^, *f*(*t*) = [*f*_1_(*t*) ⋯ *f*_*m*_(*t*)] ∈ ℝ^*m*^, with *m* < *p* ≤ *n* are the unknown inputs/uncertainties under similar assumptions, general class of nonlinear systems is defined in (35); a linear transformation [10] can always be employed to obtain the required structure for the design of the observers.

## 4. Results

For the performance evaluation of the proposed observer, we select the following active suspension system parameters of a Ford Fiesta MK2 [30] vehicle: *m*_*s*_ = 216.75 kg,  *m*_*u*_ = 28.85 kg, *k*_*s*_ = 21700 N/m, *b*_*s*_ = 1200 Ns/m, and *k*_*r*_ = 184000 N/m. The nonlinear spring stiffness, *k*_*s*nl_, and damping constant, *b*_*s*nl_, values are taken as 10% of the original linear values *k*_*s*_ and *b*_*s*_,  respectively. To design the adaptive STA, we choose the gains as *κ*_1_ = 500, *κ*_2_ = 4, *κ*_3_ = 30, *ϵ* = 0.3, and *α*_*m*_ = 4. The initial conditions for the plant and the observer were chosen as *x*(0) = $\left[\begin{array}{cccc} 0.1 & 0 & 0 & 0.01 \end{array}\right]$ and $\hat{x}(0)$ = $\left[\begin{array}{cccc} 0 & 0 & 0 & 0 \end{array}\right]$. The feedback gain for the subsystem (12) and the positive definite matrix, *Q*, satisfying (23) were computed as

(36) $$\begin{array}{c} L=\left[\begin{array}{c} 6.0259\\ 7.2397\\ 3.3748 \end{array}\right],\;\;Q=\left[\begin{array}{ccc} 0.0079 & -0.0031 & 0.0029\\ -0.0031 & 0.0108 & -0.0020\\ 0.0029 & -0.0020 & 0.0111 \end{array}\right]. \end{array}$$

**Figure 3:**
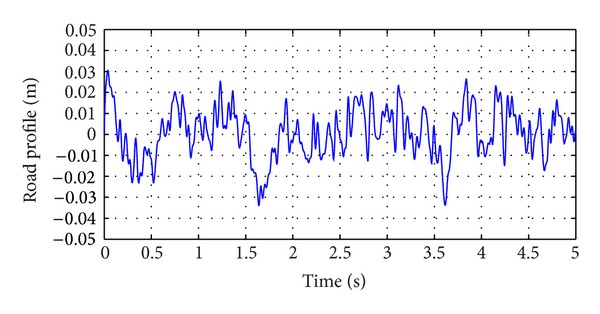
Average Class C road profile.

**Figure 4:**
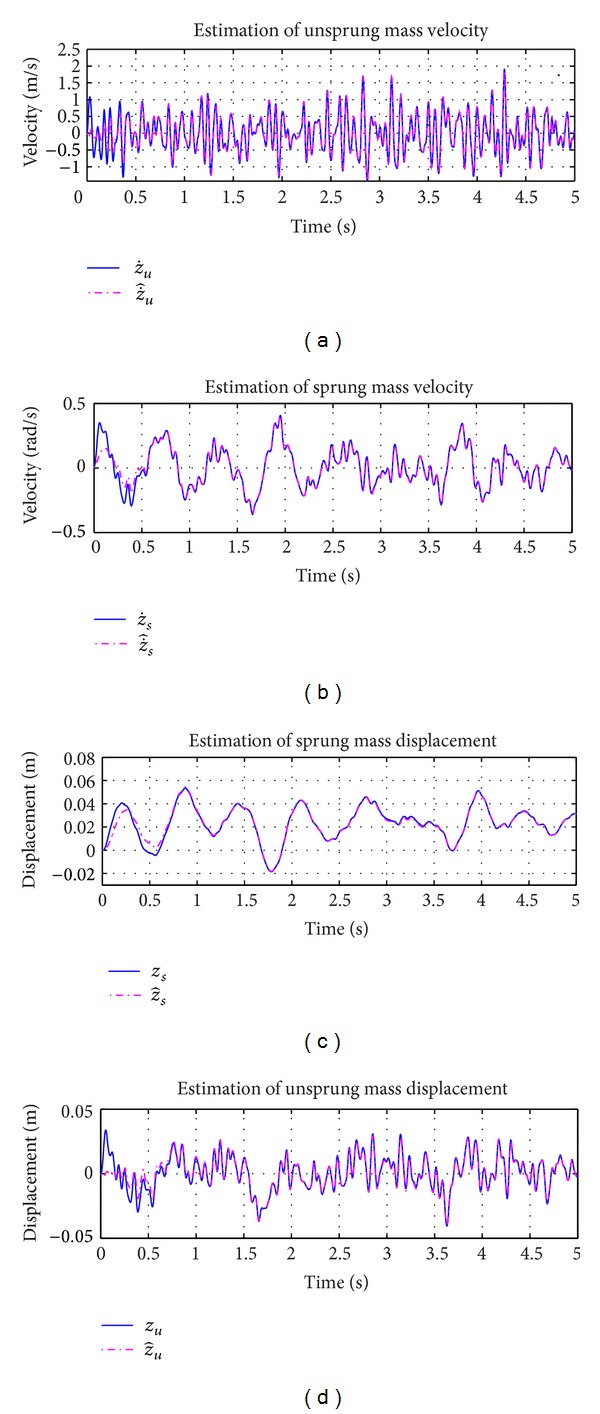
(a) Estimated unsprung mass velocity, (b) estimated sprung mass velocity, (c) estimated sprung mass displacement, (d) estimated unsprung mass displacement.

**Figure 5:**
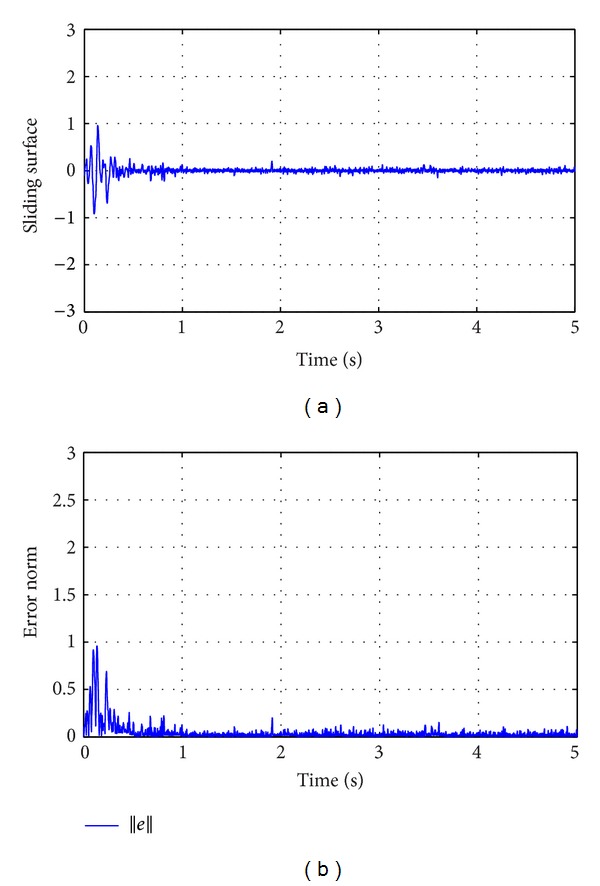
(a) Sliding surface (*e*_1_) and (b) Error norm ||*e*||.

**Figure 6:**
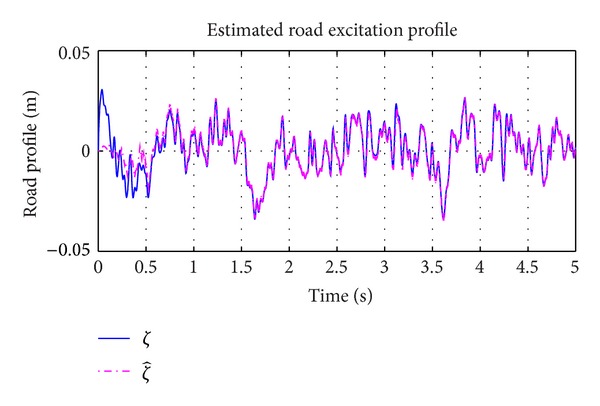
Estimation of road profile, *ζ*(*t*).

The Lipschitz constant for subsystem (12) was evaluated as *l*_*β*_ = 10.5651. The road profile model [31] can be obtained as

(37) $$\begin{array}{c} \dot{\zeta }\left(t\right)=-2\pi n_{0}v\zeta \left(t\right)+2\pi \sqrt{\sigma_{0}v}w_{0}, \end{array}$$

where *v* is the vehicle longitudinal velocity, *σ*_0_ is the road roughness coefficient, *n*_0_ is the reference space frequency, and *w*_0_ is the Gaussian white noise. With changes in road roughness coefficient keeping the longitudinal velocity of motion fixed, different excitation profiles can be obtained. The increase in roughness coefficient leads to poorer road conditions. For simulation purposes, the class C road profile was considered as an unknown input. The vehicle was considered to be travelling at a speed of 30 Km/hr with road roughness coefficient being considered as shown in Table 1. The generated road profile is shown in Figure 3. The simulation results obtained for state estimation are shown in Figure 4. The system dynamics are nonlinear and affected by the road profile, *ζ*(*t*), which is a function of white Gaussian noise. It can be deduced that the estimation of the states even under the effect of *ζ*(*t*) is good. The norm of the estimation error of the states is shown in Figure 5. In Figure 6, the unknown road excitation profile estimated with (34) is shown. A smooth estimation of the unknown road profile is obtained without any low-pass filtering.

## 5. Conclusions

In this work, an adaptive super-twisting observer was proposed for state and unknown input estimation for the active suspension system. The paper considered the nonlinear model of the active suspension system excited by the random road profile as an unknown input. Under the Lipschitz condition for the nonlinear functions, the convergence of the system errors is proven. The proposed adaptive super-twisting observer accurately estimates the road excitation profile for an average road without the use of low-pass filter.

## Figures and Tables

**Table 1:** Road roughness values classified by ISO [4, 5].

Degree of roughness σ_0_ × 10^−6^		
Road class	Range	Geometric mean
A (very good)	<8	4
B (good)	8–32	16
C (average)	32–128	64
D (poor)	128–512	256
E (very poor)	512–2048	1024
F	2048–8192	4096
